# Dickkopf-3 Contributes to the Regulation of Anti-Tumor Immune Responses by Mesenchymal Stem Cells

**DOI:** 10.3389/fimmu.2015.00645

**Published:** 2015-12-24

**Authors:** Kun-Hui Lu, Amel Tounsi, Naveen Shridhar, Günter Küblbeck, Alexandra Klevenz, Sandra Prokosch, Tobias Bald, Thomas Tüting, Bernd Arnold

**Affiliations:** ^1^Department of Molecular Immunology, German Cancer Research Center (DKFZ), Heidelberg, Germany; ^2^Laboratory of Experimental Dermatology, Department of Dermatology and Allergy, University of Bonn, Bonn, Germany

**Keywords:** Dickkopf-3, mesenchymal stem cell, anti-tumor response, T cell, tumor stroma, melanoma

## Abstract

Mesenchymal stem cells (MSCs) are known to limit immune responses *in vivo* by multiple soluble factors. Dickkopf-3 (DKK3), a secreted glycoprotein, has recently been identified as a novel immune modulator. Since DKK3 has been reported to be produced by MSCs, we investigated whether DKK3 contributes to the immune suppression of anti-tumor responses by MSCs. Whereas wild-type MSCs inhibited immune responses against two different transplantation tumors, DKK3-deficient MSCs did not affect the rejection process. Increased CD8^+^ T cell and reduced M2-type macrophages infiltration was observed in tumors inoculated together with DKK3-deficient MSCs. Thus, DKK3 could alter the composition of the tumor stroma, thereby supporting the MSCs-mediated suppression of immune responses against these tumor transplants.

## Introduction

Besides their well-known regenerative capacity, mesenchymal stem cells (MSCs) are involved in limiting undesirable immune responses, such as hypersensitivity, autoimmune disease, or alloreactivity against transplants (1–3). It has been reported that MSCs can suppress human graft-versus-host disease (4) and murine experimental autoimmune encephalomyelitis (5). Also in the contact-dependent hypersensitivity disease, systemically infused MSCs can sufficiently ameliorate local immune responses (6). Multiple clinical trials have been launched to test the capacity of MSCs to limit immunological diseases in humans (3). However, besides the potentially promising clinical applications, the immune-regulatory capacities of MSCs also support tumor growth by inhibiting T cells proliferation (7, 8), by inducing regulatory T cells (9), and by facilitating the generation of immunosuppressive M2-type macrophages (10). Multiple soluble factors, such as TGFβ (9), prostaglandin E2 (11, 12), and kynurenine (13, 14) were found to be involved in the immune-suppressive function of MSCs.

Dickkopf-3 (DKK3) is a member of an evolutionary conserved gene family encoding for five secreted glycoproteins (15, 16). It is mainly produced by tissue cells in so-called immune privileged sites, such as brain, eye, and testis (17). In addition, DKK3 expression was found in human MSCs (18, 19). We recently identified DKK3 as a novel immune-mediator limiting CD8^+^ and CD4^+^ T cell-mediated responses (20–22). In a transgenic mouse model of peripheral T cell tolerance, we showed that CD8^+^ T cells, which were neonatally tolerized toward a self-antigen exclusively expressed on keratinocytes, expressed DKK3. The function of these regulatory CD8^+^ T cells was dependent on the presence of DKK3 (20). Moreover, genetic deletion or antibody-mediated neutralization of DKK3 led to an exacerbated experimental autoimmune encephalomyelitis. This phenotype was accompanied by an increased T cell accumulation and a change in their polarization, displayed by an increase of interferon-gamma (IFNγ)-producing CD8^+^ and CD4^+^ T cells exclusively within the central nervous system (CNS) (21). We also found that DKK3 contributed to the immuno-suppressive microenvironment protecting transplanted, class-I mismatched embryoid bodies from T-cell-mediated rejection (21). DKK3 is also involved in regulating the composition of the B cell compartment (23). The development of B2 cells was impaired at the pre- and immature B cell stage in the absence of DKK3, resulting in decreased numbers of follicular B cells in adult DKK3-deficient mice. Furthermore, DKK3 limited B1 cell self-maintenance in the periphery, by decreasing the survival and proliferation of B1 cells. In addition, DKK3-deficient mice exhibited altered antibody responses and an increased secretion of the cytokine IL-10 (23).

Based on these findings, we investigated whether DKK3 is involved in MSCs-mediated immune modulation of anti-tumor responses. Our studies show that DKK3 produced by MSCs is required to maintain a tumor-promoting environment by limiting CD8^+^ T cell but supporting M2-type macrophage infiltration. Loss of DKK3 in tumor-associated MSCs results in delayed tumor growth or rejection. Thus DKK3 is an additional factor involved in the immune-suppressive function of MSCs.

## Materials and Methods

### Mice

Male C57BL/6 (B6) or RAGE-EGFP^+/+^xB6 (EGFP^+^) mice were used in different experiments. DKK3 knock-out (DKK3^−/−^) mice were mice with systemic deletion of DKK3 expression (17). Mice were bred and housed under specific pathogen-free (SPF) conditions in the central animal laboratory in German Cancer research Center (DKFZ). Animal welfare and experiments were achieved in accordance with institutional guidelines provided by the German Cancer Research Center and were approved by the Regierungspräsidium Karlsruhe (DKFZ205 and G177/12), Germany.

### Cell Culture

Selected MSCs and HCmel12 melanoma cells were cultured in complete high-glucose (4.5 mg/ml) DMEM with 10% FBS, 25 mM HEPES, and 2 mM l-glutamine. Accutase (#A6964, Sigma) was used to detach the cells. RMA-mOVA T lymphoma cells were cultured in complete RPMI1640 with the same supplements.

### Mesenchymal Stem Cells Isolation

Isolation of MSCs was based on the protocol described before (24, 25) and the manufacturer’s guides from STEMCELL Technologies by which the MesenCult™ selection medium system (#05501 and #05502, STEMCELL Technology) was produced. The femur and tibia bones were isolated from two mice scarified by CO_2_. Furs and muscles were removed by scalpels. Openings were trimmed at two ends of the bones to allow the bone marrows to be washed out by isolation buffer (1× PBS with 2%FBS and 1 mM EDTA). Washed out bone marrow was suspended in isolating buffer and kept on ice. Bones were then cracked down by scalpels into small fragments without scratching and damaging the bone membranes. Fragments of bones were soaked in digesting medium (1× PBS with 20% FBS and 0.25% collagenase type-I) in room temperature for 5 min. Soaked bone fragments were further cracked into fine pieces. The digesting buffer was then filled up to 10 ml and the containing tubes were gently vortexed in 37°C for 45 min. Treated bone pieces were suspended by directly adding isolation buffer to final volume of 30 ml and then filtered through 70 μm cell strainers. The cell strainers were washed by additional 10 ml isolation buffer. Cells from bone and bone marrow were centrifuged and the respective cell pellets were suspended in MesenCult™ selection medium. Cells were cultured in the selection medium for at least 3 weeks. After checking for expression of MSCs surface markers, the selection medium was gradually replaced by complete DMEM. When the differentiating capacities of these cells were confirmed, the MSCs were cultured only in complete DMEM for further experiments.

### Mesenchymal Stem Cells Differentiation

The differentiating medium for adipogenesis was prepared by MesenCult™ selection medium containing 5 μg/ml insulin (#I6634, Sigma), 50 μM indomethacin (#I7378, Sigma), 1 μM dexamethasone (#D4902, Sigma), and 0.5 μM IBMX (#I7018, Sigma). The medium for osteogenesis was MesenCult™ selection medium containing 20 mM β-glycerol phosphate (#G9891, Sigma), 1 nM dexamethasone (#D4902, Sigma), and 0.5 μM ascorbate 2-phosphate (#A8960, Sigma). 2 × 10^5^ MSCs were seeded in six-well microplates 1 day before the inducing of differentiation. On the day of inducing differentiation, culture medium was replaced by differentiating medium. The differentiating medium was changed every 3 days for 3 weeks. Three weeks later, the medium was removed and the cells were washed with 1× PBS twice and then fixed with 10% formalin in room temperature for 20 min. After the fixation, cells were washed with 1× PBS and then processed for staining. For staining of adipocytes, 0.5% Oil Red O (#O0625, Sigma) was dissolved in methanol. For staining of osteogenesis, 2% Alizarin Red S (#S5533, Sigma) was dissolved in distilled water in pH 4.1. Fixed cells were stained by indicated solution in room temperature for 20 min and then washed twice with 1× PBS before filled up with 2 ml 1× PBS and observed by optical microscope in 40× magnificence.

### Tumor Growth and Isolation

4 × 10^5^ cells of HCmel12 or RMA-mOVA cells mixed with equal numbers of WT or DKK3^−/−^ MSCs were washed three times with 1× PBS and then suspended in 200 μl 1× PBS. Mice were shaved and subcutaneous injection was performed to inoculate the cells at the right flank of mice. Tumor size was measured in mm and calculated by the following equation: 0.5 × (*width*)^2^ × (*length*) for approximate volume (26). For analysis of tumor-infiltrating cells and surface markers of MSCs, tumors were isolated 14 days after inoculation. Mice were sacrificed by CO_2_ and tumor nodules were taken out by scissors and forceps. Isolated tumor nodules were cut into smaller pieces and then digested by digesting buffer containing collagenase IV and DNase I in room temperature for 3 h. Digested tissues were filtered through 40 μm cell strainer and washed by complete RPMI 1640 medium. Collected cells were then ready for FACS staining.

### Flow Cytometry

To stain surface markers, harvested cells were washed twice with staining buffer (3% FBS and 0.1% sodium azide in DPBS) and stained with antibody combinations diluted in staining buffer in the dark on ice for 30 min. Stained cells were then washed twice before analysis on the BD FACSCanto™ II flow cytometry. For intracellular staining of DKK3, the fixation and permeabilization was carried out with the buffer sets (#560409, BD and 88-8824-00, eBioscience) according to manufacturer’s instructions.

### Western Blotting

Mesenchymal stem cells or RMA-mOVA cells were lysed directly on the 10 cm petri dishes or in pellet by M-PER™ mammalian protein extraction reagent (#78501, Thermo Fischer Scientific) containing cocktails of protease (#04693159001, Roche Life Science) and phosphatase inhibitors (#5872, Cell Signaling Technology) after being washed by 1× PBS for two times. Cell debris was removed by 13000 rpm. Centrifugation was done for 10 min. Supernatants containing proteins of interest were analyzed by Western blotting by using mini-gel system from BioRad for the SDS-PAGE. Acrylamide gels were prepared in 10% of acrylamide concentration. Proteins were transferred by Trans-Blot^®^ SD Semi-Dry Transfer Cell and detected by specific primary antibodies and HRP-conjugated antibodies. The results were visualized by HRP substrates (#WBKLS0100, Merck Millipore) and optical films according to the manufacturer’s guides.

### Antibodies

For analysis by flow cytometry: anti-mouse CD3ϵ PerCP-eFluor^®^710 (clone 17A2, #46-0032-82 eBioscience); anti-mouse CD4 Pacific Blue™ (clone RM4-5, #100531 BioLegend); anti-mouse CD8α APC-Cy7 (clone 53-6.7, #100714 Biolegend); anti-mouse CD11b PE-Cy7 (clone M1/70, #101216 Biolegend); anti-mouse CD29 biotinylated (clone HMβ1-1, #102203 Biolegend); anti-mouse/human CD44 PE-Cy7 (clone IM7, #103030 Biolegend); anti-mouse CD105 biotinylated (clone MJ7/18, #120404 Biolegend); anti-mouse CD206(MMR) Brilliant Violet 421™ (clone C068C2, #141717, Biolegend); anti-mouse F4/80 AlexaFluor^®^ 647 (clone BM8, #123122 Biolegend); anti-rabbit IgG HRP-linked Antibody (#7074 Cell Signaling Technology); Cofilin (D3F9) XP^®^ Rabbit mAb (#5175 Cell Signaling Technology); anti-DKK3 antibody (20).

### Statistics

ANOVA followed by the Holm-Šídák approach to multiple comparisons was applied throughout the studies except for the analysis on phenotypes of MSCs, for which the unpaired *t*-test was applied.

## Results

### DKK3 Expression by MSCs is Essential for Their Suppressive Activity of Anti-Tumor Responses

**Figure 1:**
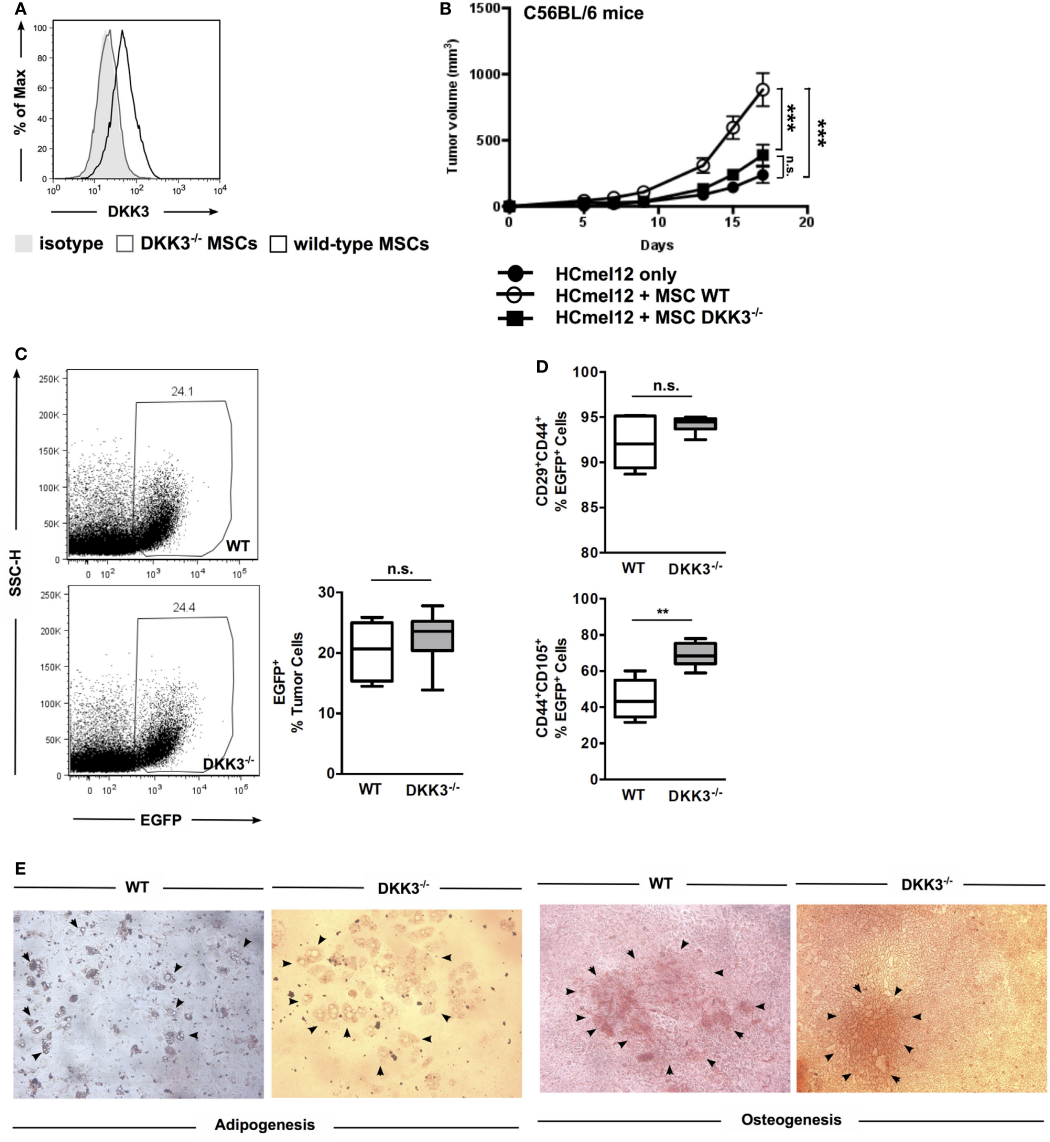
**DKK3 expression by MSCs is essential for their suppressive activity of anti-tumor responses**. **(A)** DKK3 expression was detected in wild-type mesenchymal stem cells by flow cytometry. **(B)** 4 × 10^5^ HCmel12 cells with or without equal number of mesenchymal stem cells (MSCs) were inoculated subcutaneously into B6 mice. Better growth in size and speed was observed when tumor cells were inoculated together with DKK3-competent MSCs (○, *n* = 13) in comparison with DKK3^−/−^ MSCs (●, *n* = 13) or without any MSCs (■, *n* = 13) (*p* < 0.0001 or *p* < 0.0001). No difference was observed between the growth of tumor with DKK3^−/−^ MSCs and without any MSC (*p* = 0.1175). Two independent experiments were performed. ANOVA followed by the Holm-Šídák approach to multiple comparisons was applied. **(C)** 4 × 10^5^ EGFP-positive wild-type MSCs (*n* = 7) or DKK3^−/−^ MSCs (*n* = 7) were subcutaneously inoculated together with equal number of HCmel12 cells into B6 mice. Fourteen days later, there was no difference in the percentage of wild-type and DKK3^−/−^ EGFP^+^ cells recovered from the HCmel12 tumor nodules (*p* = 0.1309). **(D)** When identifying tumor-associated MSCs with mesenchymal stem cell marker in HCmel12 tumor, no change was observed on CD29^+^CD44^+^ population (*p* = 0.1053). However, CD44^+^CD105^+^ cells were significantly increased in DKK3^−/−^ MSCs (*p* = 0.0014). Two independent experiments were performed. Unpaired *t*-test was applied between two groups. **(E)** *In vitro* differentiation showed that DKK3^−/−^ MSCs maintained competent differentiation capacity as their wild-type counter part in adipogenesis and osteogenesis. The solid arrows indicate adipocytes or calcified areas.

First, we confirmed that DKK3 expression can be detected in wild-type (WT) MSCs and is absent in MSCs derived from bone marrow of DKK3-deficient mice (DKK3^−/−^ MSCs) (Figure 1A). To test the immunosuppressive activity of MSCs, the HCmel12 melanoma transplantation tumor model was employed. Growth of these tumor cells in C57BL/6 mice was significantly accelerated when tumor cells were injected together with WT MSCs in comparison to tumors inoculated without any MSCs or with DKK3^−/−^ MSCs (both *p* < 0.0001) (Figure 1B). No difference was observed between the growth of tumor with DKK3^−/−^ MSCs and without any MSC (*p* = 0.1175) (Figure 1B). This suggested that DKK3 might be essential for MSCs to promote tumor growth.

To exclude the possibility that the observed decrease in tumor growth in the presence of DKK3^−/−^ MSCs was due to poorer survival or loss of MSC characteristics *in vivo*, we compared the viability and MSC phenotypes of WT and DKK3^−/−^ MSCs. MSCs were derived from bone marrow of EGFP^+^ C57BL/6 or EGFP^+^ DKK3^−/−^ mice and inoculated into C57BL/6 mice together with HCmel12 melanoma cells (Figures 1C,D). On day 14, single cell suspensions of the isolated tumors were generated and the respective EGFP^+^ cells identified. The percentage of DKK3-sufficient and -deficient MSCs within the respective tumors was comparable (Figure 1C). Antibody staining for the MSC marker proteins CD29, CD44, and CD105 and flow cytometry was used to determine the phenotype of the isolated MSCs (Figure 1D). Expression of CD29 was similar for both types of MSCs isolated from the tumors while the CD105^+^ cells were even increased in DKK3^−/−^ MSCs (*p* = 0.0014). Moreover, DKK3^−/−^ MSCs are as capable as WT MSCs for undergoing *in vitro* adipogenesis or osteogenesis (Figure 1E). Thus, DKK3^−/−^ MSCs were normally persistent and maintained their phenotypes within the tumor transplants.

### DKK3 Expression by MSCs Contributes to Modulating the Composition of Tumor-Infiltrating Immune Cells

**Figure 2:**
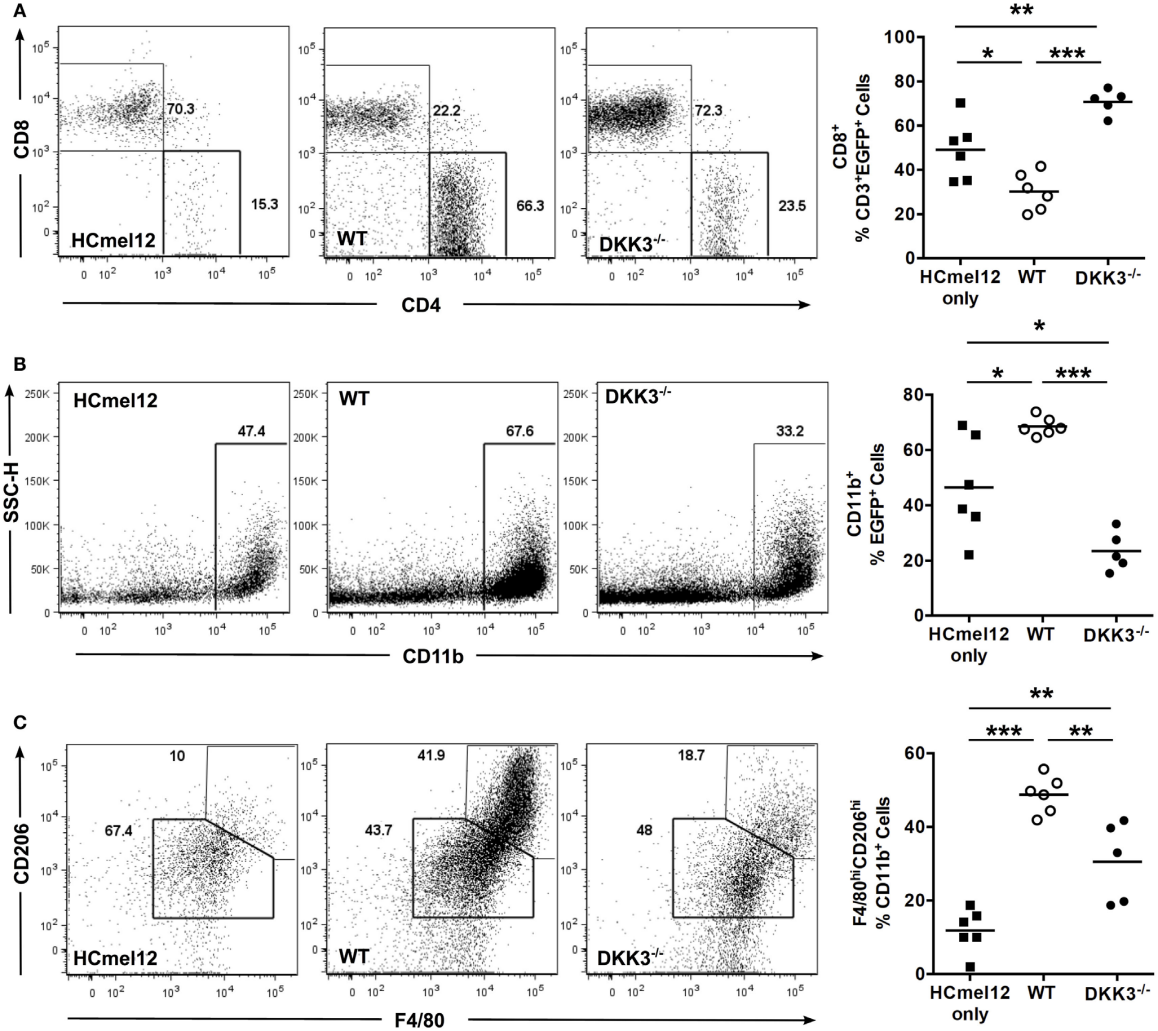
**DKK3 expression by MSCs contributes to modulating the composition of tumor-infiltrating immune cells**. **(A)** 4 × 10^5^ HCmel12 melanoma tumor cells only (■, *n* = 6) or with equal number of MSCs (○, wild-type, *n* = 6; ●, DKK3^−/−^, *n* = 5) were inoculated subcutaneously into EGFP^+^ mice with B6 background. Fourteen days later, tumor nodules were isolated and the infiltrating EGFP^+^ cells were analyzed by flow cytometry. A significant reduction in the percentage of infiltrating CD8^+^ T cells was detected in tumors with wild-type MSCs in comparison with tumors without any MSC (*p* = 0.0142). In contrast, an increased infiltration of CD8^+^ T cells was observed for tumors with DKK3^−/−^ MSCs in comparison with tumors with wild-type MSCs (*p* < 0.0001) or with tumors without any MSC (*p* = 0.0076). **(B)** A higher percentage of CD11b^+^ cells was found in wild-type MSCs-associated tumors in comparison with the tumor only group (*p* = 0.0136), whereas tumor-infiltrating CD11b^+^ cells were significantly decreased in tumors containing DKK3^−/−^ MSCs in comparison with tumors containing wild-type MSCs (*p* < 0.0001) and in comparison with HCmel12 tumors without any MSC (*p* = 0.0141). **(C)** M2-type macrophages were defined as F4/80^hi^CD206^hi^ cells out of CD11b^+^ cell population. A higher percentage of M2-type macrophages was found in tumors with wild-type MSCs in comparison to tumors containing DKK3^−/−^ MSCS (*p* = 0.0033) and with tumors without any MSC (*p* < 0.0001). The level of M2-type macrophages remained higher in tumors containing DKK3^−/−^ MSCs in comparison with tumors without any MSC (*p* = 0.0025). Two independent experiments were performed. ANOVA followed by the Holm-Šídák approach to multiple comparisons was applied for the analysis.

Next, we analyzed tumor-infiltrating immune cells of HCmel12 tumors 14 days after inoculation into EGFP^+^ C57BL/6 mice. The percentage of CD8^+^ T cells among CD3^+^ EGFP^+^ cells was decreased in tumors containing WT MSCs in comparison with tumors inoculated without any MSCs (*p* = 0.0142). In contrast, tumor-infiltrating CD8^+^ T cells were significantly increased in tumors containing DKK3^−/−^ MSCs in comparison to tumors inoculated with WT MSCs or tumors without any MSCs (*p* < 0.0001 and 0.0076) (Figure 2A). Furthermore, a higher percentage of CD11b^+^ cells among EGFP^+^ cells was found in tumors with WT MSCs in comparison with tumors with DKK3^−/−^ MSCs or without any MSCs (*p* < 0.0001 and *p* = 0.0136) (Figure 2B). F4/80^hi^CD206^hi^ M2-type macrophages were significantly expanded among CD11b^+^ cells in tumors with WT MSCs in comparison with tumors with DKK3^−/−^ MSCs or without any MSCs (*p* = 0.0033 and *p* < 0.0001) (Figure 2C). Thus, DKK3 expression by MSCs contributed to modulating the tumor microenvironment by limiting CD8^+^ T cell infiltration and promoting M2-type macrophage appearance.

### DKK3 Expression Exclusively by MSCs is Sufficient to Limit T Cell Responses Against the Strongly Antigenic RMA-mOVA Tumor

**Figure 3:**
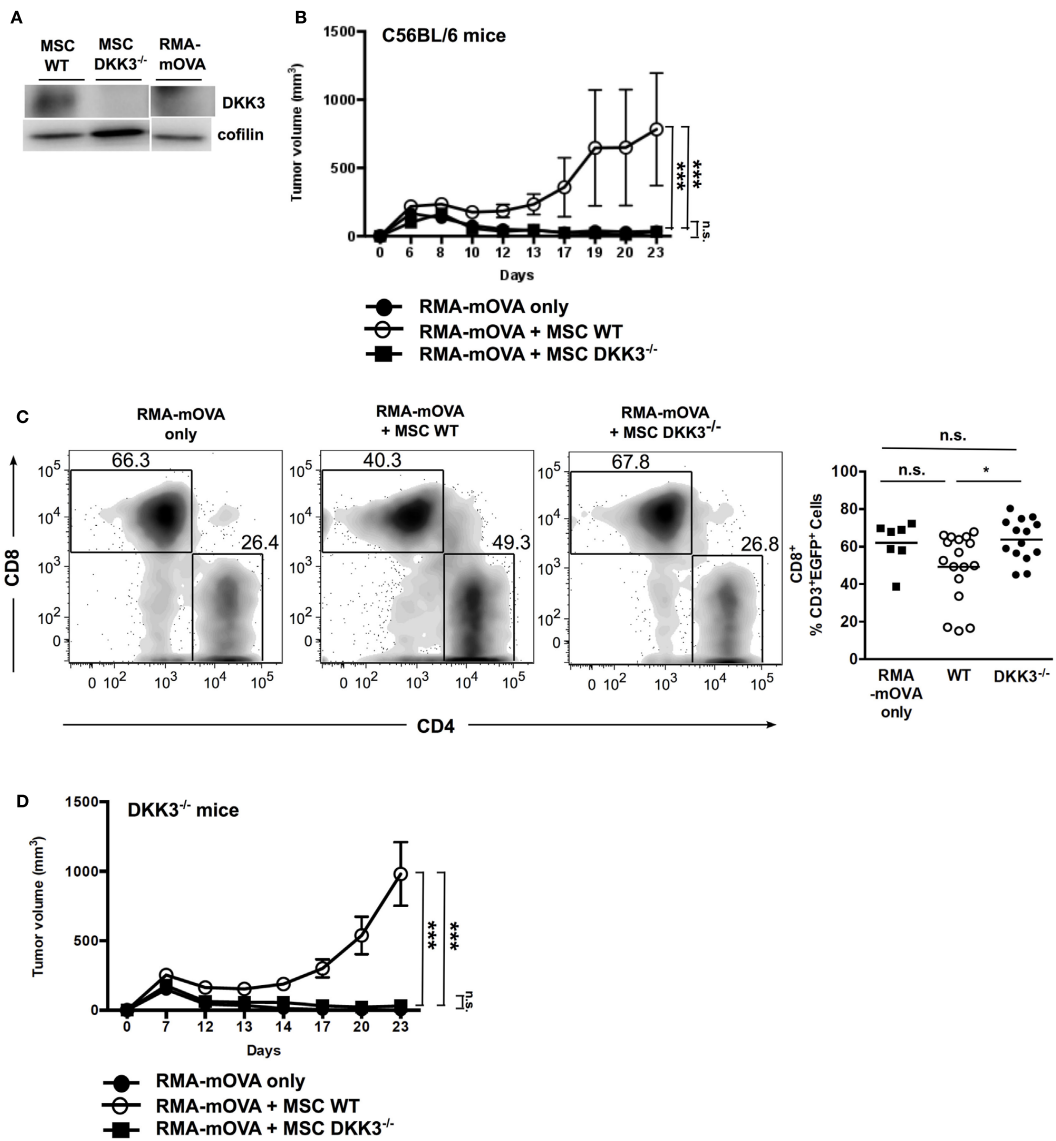
**DKK3 expression exclusively by MSCs is sufficient to limit T cell responses against the strongly antigenic RMA-mOVA tumor**. When inoculating 4 × 10^5^ RMA-mOVA tumor cells, which did not express autologous DKK3 **(A)**, along with equal cell number of MSCs, **(B)** tumors with wild-type DKK3-competent MSCs (○, *n* = 6) kept significant growth without rejection in comparison with tumors containing DKK3-deficient MSCs (■, *n* = 6) or without any MSC (●, *n* = 6) (*p* < 0.0001 or *p* < 0.0001). No difference in tumor growth could be distinguished between tumors with DKK3^−/−^ MSCs and tumors without any MSC (*p* = 0.8516). **(C)** By inoculating RMA-mOVA cells with either wild-type (●, *n* = 17) or DKK3^−/−^ MSCs (■, *n* = 14) or without any MSC (■, *n* = 7) into EGFP^+^ B6 mice, tumor-infiltrating immune cells in the isolated tumor nodules were identified as EGFP^+^ cells after 14 days. Tumor-infiltrating CD8^+^ T cells were significantly increased in DKK3^−/−^ MSC-inoculated tumors in comparison with tumors with wild-type MSCs (*p* = 0.0314). No difference was observed between tumors only and tumors containing either WT or DKK3^−/−^ MSCs (*p* = 0.1190 or *p* = 0.8186). Two independent experiments were performed. **(D)** In DKK3^−/−^ mice, growth of RMA-mOVA tumors was only observed when tumor cells were co-injected with DKK3-competent MSCs (○, *n* = 6), which was in contrast to tumors inoculated with DKK3-deficient MSCs (■, *n* = 6) or without any MSC (●, *n* = 6) (*p* < 0.0001 or *p* < 0.0001). No difference in tumor growth was observed between tumors harboring DKK3^−/−^ MSCs and tumors without any MSC (*p* = 0.4166). ANOVA followed by the Holm-Šídák approach to multiple comparisons was applied.

To investigate whether DKK3 produced by MSCs could also limit the growth of a strongly antigenic tumor, we used the RMA-mOVA tumor cell line. These tumor cells express membrane-bound Ovalbumin (OVA) and elicit a strong cytotoxic T cell response in C57BL/6 mice (27). Furthermore, these tumor cells do not express DKK3 (Figure 3A). RMA-mOVA tumors inoculated together with WT MSCs were accepted by C57BL/6 mice, whereas RMA-mOVA tumors without MSCs or with DKK3^−/−^ MSCs were rejected (Figure 3B). Furthermore, we observed that RMA-mOVA tumor-infiltrating CD8^+^ T cells were significantly increased in tumors containing DKK3^−/−^ MSCs in comparison to tumors containing WT MSCs (*p* = 0.0314) (Figure 3C), as we had already shown for the HCmel12 tumors (Figure 2A). Finally, we asked whether the DKK3 produced by WT MSCs is responsible for the observed effect. Therefore, the RMA-mOVA tumor cells, which do not express DKK3, were injected into DKK3-deficient mice. Again, only the RMA-mOVA tumors injected together with WT MSCs could grow, while the tumors without MSC or with DKK3^−/−^ MSCs were rejected (Figure 3D). This indicated that the DKK3 produced by MSCs, but neither by the tumor cells nor by the environment, might be capable of down-modulating the immune response against this transplanted tumor.

## Discussion

Here, we demonstrate that DKK3 is an additional important soluble factor for MSCs to regulate immune responses in the tumor microenvironment. Loss of DKK3 impaired the immune-suppressive capacity of MSCs resulting in rejection of transplanted tumors, which were accepted in the presence of DKK3-sufficient MSCs. Increased CD8^+^ T cell and reduced M2-type macrophages infiltration were observed in tumors containing DKK3^−/−^ MSCs.

Many studies have demonstrated that MSCs can promote tumor growth and formation of metastasis, whereas other studies reported that MSCs suppress tumor progression (28). Presently, there is no simple explanation for these conflicting findings. Further investigations are needed for a better understanding of the interactions between MSCs and cells present in a tumor, especially because of the therapeutic potential of MSCs. Therefore, we have chosen two models of tumor transplants whose progression is supported by MSCs to investigate the role of DKK3. Although DKK3^−/−^ MSCs were normally persistent within the tumor transplants, they did not accelerate tumor growth in C57BL/6 mice as WT MSCs did. We observed an increase in CD105^+^ cells among the DKK3^−/−^ MSCs. It was recently reported that CD105^−^ MSCs represent an independent subpopulation (29). The CD105^+^ and CD105^−^ mMSC subpopulations had similar growth potential, but varied in their immunoregulatory properties. Interestingly, CD105^−^ mMSCs suppressed the proliferation of CD4^+^ T cells more efficiently in comparison with CD105^+^ mMSCs. Therefore, it is possible that losing DKK3 promotes the differentiation and/or the survival of the CD105^+^ MSC subpopulation and leads to a reduction in the suppressive capacity of DKK3^−/−^ MSCs. It had been reported that DKK3 produced by human MSCs could decrease cell cycle progression of tumor cells *in vitro* (19). Presently, we cannot judge whether or not DKK3 can directly modulate the growth of the used tumor transplants *in vivo*. However, we would like to conclude that DKK3 is contributing to the immune-suppressive capacity of MSCs because DKK3 could reduce tumor infiltration by CD8^+^ T cells, which had been shown to contribute to the rejection of RMA-mOVA tumors (28). This is in concordance with our recent reports in which DKK3 could limit CD8^+^ and CD4^+^ T cell-mediated responses (20–22).

Molecular mechanisms of DKK3 functions are still unknown. So far, no surface receptor for DKK3 has been identified in mice or humans. It has been reported that recombinant human DKK3 could be internalized by induced pluripotent stem cell-derived embryoid bodies via endocytosis (30). Such an internalization process may explain how DKK3 could interact with the cytoplasmic protein b-TrCP, thereby acting as a negative regulator of Wnt signaling (31). Therefore, it is possible that the immune-modulatory function of DKK3 may be based at least in part on its modulation of the Wnt pathway, especially as Wnt signaling is known to influence T-cell effector function (32) and linage commitment (33). However, the distinct cellular and molecular mechanisms through which DKK3 mediates its function warrant further investigation.

Dickkopf-3 is also known as “REIC” (Reduced Expression in Immortalized Cells), as it was discovered in transcriptome screening of primary tumors (34). Since then numerous reports proposed that DKK3 can act as a tumor suppressor. DKK3 has been claimed to be downregulated in a broad range of cancers, such as non-small cell lung cancer (35), breast cancer (36, 37), gastric cancer (38), and melanoma (39), and this reduced expression was correlated with lower survival rates of patients from the respective cancer types. In contrast, deletion at the DKK3 locus was related with lower lymph node metastasis and better prognosis in head and neck squamous cell carcinomas (40), indicating that DKK3 in this type of cancer is apparently not acting as a tumor suppressor but may more likely function as an immune modulator, as we have shown here and in previous studies (20–22). Thus the exact role and possible application of DKK3 may be dependent on the type of tumor and the cellular context.

Overall, our studies provide evidences that DKK3 is contributing to the immune-suppressive function of MSCs and may explain the underlying mechanism in those cancer types that show better prognosis associated with impaired DKK3 expression. In addition, our findings strongly suggest careful considerations whether or not DKK3 may be used as a therapeutic “agent” or “target” in the treatment of cancers (41).

## Author Contributions

KL, AT, NS, GK, AK, and SP performed the experiments; TB, TT, and BA guided deep discussion and experimental design; KL and BA prepared the manuscript; and BA supervised the research progress.

## Conflict of Interest Statement

The authors declare that the research was conducted in the absence of any commercial or financial relationships that could be construed as a potential conflict of interest.
